# Drug Resistance and Novel Therapies in Cancers in 2019

**DOI:** 10.3390/cancers13040924

**Published:** 2021-02-23

**Authors:** Zhixiang Wang

**Affiliations:** Signal Transduction Research Group, Department of Medical Genetics, Faculty of Medicine and Dentistry, University of Alberta, Edmonton, AB T6G 2H7, Canada; zwang@ualberta.ca

After the successful launch in the second half of 2018 by *Cancers*, the topic collection “Drug Resistance and Novel Therapies in Cancers” experienced its productive first full year in 2019. 

In 2019, a total of 34 cutting-age research articles and comprehensive review papers had been published under this topic collection. The 26 original research articles covered 13 different cancer types including melanoma [1,2,3,4], breast cancer [5,6,7,8], colorectal cancer (CRC) [9,10,11], hepatocellular carcinoma [12,13,14], glioblastoma [15,16], non-small cell lung cancer (NSCLC) [17,18], prostate cancer [19], ovarian cancer [20], pleural mesothelioma [21], cervical cancer [22], Leukemia [23], adenoid cystic carcinoma [24], and vulvar cancer [25]. While these studies looked at a broad range of cancer therapies including cytotoxic chemotherapy [7,8,11,16,20], electrochemotherapy [25], radio therapy [2,4,18], proton beam therapy [24], and oncolytic virus therapy [6], the most studied are targeted therapies with various means. The cellular process being targeted include glucose metabolism [19], cell signaling [5], endoplasmic reticulum (ER) stress [21], autophagy [12], angiogenesis [10], DNA repair [4,6], apoptosis [11], and microRNA [10,26]. The molecules being targeted include HER2 [5], VEGF [10], p53 [4,13,18], CDK [14], BCR-ABL1 and PAK1/2 [23], PARP1/HMGB1 [12], mir-221 [26], miR-31-5p [10], TIMP-1 [17], Fox3a [8], and FOXC1 [10].

Most of these research articles are well cited, which indicates the significance of their findings. The study regarding the role of FOXC1 regulation of miR-31-5p in oxaliplatin resistance by Huang’s group was cited 13 times within a year of publication [10]. Oxaliplatin (OXA) is currently used in first-line chemotherapy to treat stage III and stage IV metastatic CRC. In this research, the authors studied the role of miRNA in the drug resistance mechanism in an OXA-resistant CRC cell model and in a xenograft animal model. They showed that the upregulated expression of FOXC1 and miR-31-5p inhibited LATS2 expression, which led to OXA resistance. They further showed that the knockdown of miR-31-5p resulted in cancer cell apoptosis, decreased cell proliferation, and enhanced chemosensitivity. These results revealed a novel drug-resistance mechanism in which miR-31-5p and its transcription factor regulate cancer cell growth and apoptosis by targeting LATS2. 

In another well-cited piece of research, Singh et al studied therapeutic potential of a natural flavonoid morin hydrate against cisplatin-induced toxicity by using the HepG2DR multi-drug resistant cell line [12]. They showed that dysregulated expression of PARP1 confers cisplatin-resistance via autophagy activation in HepG2DR cells. Morin hydrate inhibits cisplatin-mediated autophagy induction, resulting in increased susceptibility of HepG2DR cells to cisplatin cytotoxicity. This research suggests that the combination of morin hydrate with cisplatin may be a promising therapeutic strategy to enhance the efficacy of conventional chemotherapeutic drugs.

In another well-cited novel study, Xu et al explored novel target for treating malignant pleural mesothelioma (MPM), a rare malignant cancer [21]. They found that ER stress and the adaptive unfolded protein response (UPR) signaling are characteristically deregulated in MPM. Consequently, pharmacological perturbation of ER stress/UPR axis by HA15, an agent that induces persistent proteotoxic stress in the ER, selectively suppresses the viability of MPM cells. Their findings revealed that HA15 could be used to target ER stress signaling and serve as a promising agent to treat patients with MPM.

In a study aiming to understand the molecular mechanisms underlying the action of pertuzumab and its combination with trastuzumab, Nami et al [5] showed that pertuzumab had no significant effect on HER2 homodimerization; however, trastuzumab increased HER2 homodimerization. Pertuzumab, but not trastuzumab, abrogated the effect of HER2 overexpression on cell cycle progression, whereas trastuzumab abolished the inhibitory effect of HER2 on apoptosis. These results suggested that the clinical effects of pertuzumab may mostly, through the inhibition of HER2 heterodimers, rather than HER2 homodimers and that pertuzumab binding to HER2, may inhibit non-canonical HER2 activation and function in non-HER-mediated and dimerization-independent pathway(s).

Among the three studies regarding p53, one study showed that p53 DNA binding domain mutations predicted progression-free survival of bevacizumab therapy in metastatic CRC [10]. Another study assessed the potential benefit of adding radiotherapy to BRAF-mutated melanoma cells under a combined p53 reactivation and MAPK inhibition and showed that the combination of BRAF inhibition together with p53 reactivation significantly enhanced the radiosensitivity of BRAF-mutant melanoma cells [4]. The third study showed that SLMP53-2 restores the function of mutant p53 through Hsp70 in hepatocellular carcinoma [13].

There were eight comprehensive reviews published in 2019 and most of them are well cited. One review article aimed to discuss the PI3K/AKT/mTOR and CDK4/6 pathways in endocrine resistant HR+/HER2− metastatic breast cancer [27]. In this review, the authors analyzed the PI3K/AKT/mTOR and CDK4/6 pathways and their roles in endocrine resistant metastatic breast cancer. The review focused on the new therapeutic agents developed and the roles of these agents in overcoming endocrine resistance. The central nervous system (CNS) is considered as a sanctuary site, protected from systemic chemotherapy by the meninges, the cerebrospinal fluid (CSF) and the blood–brain barrier (BBB). In one review, the authors described the different strategies developed to improve delivery of antineoplastic agents into the brain in primary and metastatic CNS tumors [28]. DNA damage leads to cancer development and RAD52 has a crucial role in repairing DNA damage. In the review regarding RAD52 functions in homologous recombination and in cancer therapy, Nogueira et al discussed recent reports regarding the role of up- and down-regulation of RAD52 in carcinogenic processes and in targeting RAD52 to improve cancer therapy efficacy [29]. Two reviews focused on CRC. One reviewed the current literature on angioregulatory miRNAs in CRC [30] and the other one is about the tumor microenvironment in CRC therapy [31]. The other three reviews discussed resistance in various cancers. One focused on overcoming drug resistance by pressurized intraperitoneal aerosol chemotherapy (PIPAC) [32], one reviewed therapeutic challenges in managing cisplatin-resistant ovarian germ cell tumors [33], and one examined the resistance to systemic agents in renal cell carcinoma [34].
